# Porosome: a membrane microdomain acting as the universal secretory portal in exocytosis

**DOI:** 10.15190/d.2014.21

**Published:** 2014-09-23

**Authors:** Mircea Leabu, Cristina Mariana Niculite

**Affiliations:** University of Medicine and Pharmacy "Carol Davila", and "Victor Babes" National Institute of Pathology, Bucharest, Romania

**Keywords:** porosome, membrane fusion, cell secretion, exocytosis, membrane traffic

## Abstract

Most, if not all, cells in the organism, at least in some period of their lifetime, secrete materials that are produced within the cell. Cell secretion is a phenomenon requiring membrane fusion at a specialized plasma membrane structure called the ‘porosome,’ which allows the material stored within secretory vesicles to be delivered to the cell’s exterior environment. This is achieved when the secretory vesicles fuse at the base of the porosome complex, establishing a fusion pore or fluid continuity between the vesicle interior and the cell’s exterior. Besides cell secretion, membrane fusion is necessary for intracellular membrane traffic and vesicular transport from one endomembrane bound structure to another. In addition to cell secretion, membrane fusion is necessary for intracellular membrane trafficking and vesicle transport from one intracellular membrane to another. We suggest that the debate about whether to use the term ‘porosome’ or ‘fusion pore’ to describe this process is unnecessary, since both of these terms are useful in describing aspects of the last event of cell secretion, namely exocytosis. In this review, we will summarize the information related to the discovery of the porosome, a universal secretory portal for exocytosis, and discuss porosome molecular organization and function. Finally, we will develop the notion that the porosome is a specialized plasma membrane microdomain.

## SUMMARY

1. *Introduction*
2. *Corner stones in the history of cell secretion and membrane fusion*
3. *Ultrastructure of the porosome*
4. *Molecular organization of the porosome*
5. *Porosome or fusion pore, or porosome and fusion pore*
6. *Porosome: a membrane microdomain *
7. *Concluding remarks*

## 1. Introduction

Membrane fusion is a ubiquitous biological event in the life of a cell. It is required for movement of materials from outside to inside cells and its converse, as well as for trafficking of membranes and soluble materials within cells. Indeed, membrane fusion is considered a “*sine qua non*” phenomenon in secretory pathways. Therefore, deciphering the mechanism of membrane fusion became a challenging task for cell biologists. The first review on this topic appeared in 1973^1^. Post and Allison^1^ pointed out the significance of membrane fusion in exocytosis, for both digestion and transport, although the term transcytosis had not yet been introduced. They also stressed the importance of turnover and redistribution of intracellular membranes and exocytosis. They related that “the major significance of the storage of secretory products within vesicles lies not so much in the storage process, […] but in the process by which these products are released [in exquisite precision, we can add] from the cell by exocytosis”. They also mentioned that “exocytosis offers a further advantage over other methods of secretion in that the stimulus for the release of the secretory product can act directly on the plasma membrane which is also the site of fusion and release. This enables the secretion of material by exocytosis to be restricted to specific regions of the plasma membrane by means of differences in the ability of various areas of the plasma membrane to respond to the release stimulus and by differences in the capacity of regions of the plasma membrane to fuse with the secretory granule membrane”^1^. Moreover, Post and Alison intuitively suggested that “exocytotic activity involving fusion of secretory granules with the plasma membrane would be expected to produce a significant increase in the surface area of the plasma membrane unless mechanisms were available to compensate for this increase”^1^.

At the present time, we know that the diversity of exocytotic mechanisms is related to the destiny of vesicle membranes, whether they undergo events such as “full fusion”, “kiss-and-run” or “kiss-and-coat” processes^2,3^. In addition, any dispute about membrane fusion as an event governed by the membrane lipids or controlled by membrane proteins is over. We know that many types of membrane components, both lipid and protein, are important in the act of membrane fusion. For example, the best described proteins that act during membrane fusion are SNAREs (Soluble *N*-ethyl-maleimide-sensitive factor attachment protein receptor), and they are distributed on specific membrane microdomains with a cholesterol-enriched composition^4^. However, other proteins contribute to the precision in time and space of membrane fusion^5^. Here we will discuss an important membrane nanostructure that is organized as a membrane microdomain called a porosome. The porosomes are important in secretory vesicle docking and fusion at the target membrane where exocytosis occurs.

## 2. Corner stones in the history of cell secretion and membrane fusion

Secretion is a cellular function that is essential for tissue and organ biogenesis and homeostasis as well as for social interactions with other cells. The term cell secretion was apparently first used (according to a Pub Med search) in 1949^6^. However, searching on Google Academic, we find a paper, using “cell secretion”, in 1911^7^, which reports an abnormal mucous secretion. As a matter of fact, it is possible to be surprised regarding the first use of the term after a search in a well documented library, but it is not our goal here to make a rigorous historical investigation. A significant moment in the transition from intuition to proof was made in 1964 by a paper published by Lucien Caro and George Palade^8^. These authors reported for the first time the pathway of cell secretion in pancreatic acinar cells^8^. A nice portrayal of this first great step in our knowledge of cell secretion can be found in Palade’s Nobel lecture^9^. Almost 50 years have passed before cell biologists could decipher at the molecular level the pathway followed by a secreted protein from its biosynthesis to its release to the outside of the cell.

At the time that Caro and Palade were undertaking their ultrastructural observations there was no detailed information at the molecular level on the mechanisms involved in this complex cellular event, except that it involved several organelles (ribosomes, endoplasmic reticulum, Golgi apparatus), as well as other cellular ultrastructures such as secretory vesicles and cell membrane.

In time, a new concept appeared that was necessary to describe the mechanism of cell secretion, namely membrane fusion. Nothing contained in a vesicle inside the cell can be exocytosed without fusion of the secretory vesicle membrane with the cell membrane. The first apparent use of the term “membrane fusion” dates to 1962 and is related to fertilization in plants and animals^10^, whereas use of the term fusion in relationship to cell secretion was first mentioned in 1973^11^. This occurred in spite of a theory on membrane fusion that was advanced in 1971^12^. This phenomenon was presented as an important event in cell biology, even though only two cellular processes were pointed out to support the idea: endocytosis needed for directing engulfed material to digestion in lysosomes, and exocytosis for transporting materials from inside the cell to the extracellular space. According to this theory, “membrane fusion reaction is considered in four stages: membrane contact; induction; fusion; and stabilization”^12^. This contribution was based on the fact that “very few studies [are] concerned directly with the basic mechanisms involved in this phenomenon”, but some events related to membrane fusion are mentioned. For example, the authors stated that “under appropriate conditions changes in the electrostatic potential created by the closed approximation of membranes (stage one) will induce the membrane changes required for the remaining three stages”^12^. They considered various factors that influence each stage of the membrane fusion, while the significance of membrane fusion in cell biology and objections to existing theories of fusion are discussed briefly. This was a mainly intuitive approach based on only a few studies on the topic.

In 1987, three publications presented another concept related to membrane fusion in cell secretion, the fusion pore^13-15^. In one of these papers, the authors suggested that the elements of the vesicle membrane do not mix with the elements of the plasma membrane, indicating that the stabilization stage is not necessary. Instead, the fusion pore was proposed as a reversible event^12^. Zimmerberg^14^ pointed out that differences between fusion events studied with model lipid membranes compared to plasma membranes could be explained by the presence of membrane proteins. We now know that plasma membrane proteins act to control and regulate membrane fusion events in exocytosis. At the time the fusion pore was considered as a membrane hole opened by the fusion of an exocytotic membrane vesicle with the plasma membrane.

The concept of specific membrane docking before fusion and the presence of a cell membrane structure that facilitates docking was discussed by Bhanu Jena^16,17^. He coined the term “porosome” to describe this structure, which was inferred from ultrastructural studies. The porosome was described as “a cup-shaped lipoprotein-containing basket-like structure at the plasma membrane where secretory vesicles dock and then fuse to release vesicular contents^16,17^. The structure of this element has been described as a circular array of t- and v-SNARE proteins (target- and secretory vesicle SNAREs) that must dock at the inner plasma membrane surface before fusion of the vesicle and plasma membranes can occur^16,17^.

## 3. Ultrastructure of the porosome

The introduction of Atomic Force Microscopy (AFM) was useful in delineating the ultrastructure of the porosome. Initially pancreatic acinar cells were used to study porosome ultrastructure^16^. The nanometer-scale resolution of AFM permitted imaging possibilities not offered by earlier light and electron microscope studies on exocytosis, allowing for the first time visualization of live membrane structures at a scale that could be used to visualize the porosome structure. AFM images are created by utilizing force spectroscopy. A sharp tip – located at the end of a cantilever – which scans the surface of the sample, whose displacement in 3D is monitored to obtain the three-dimensional surface topology of the object. This force is maintained constant by a feedback mechanism^18^.

AFM studies on live pancreatic acinar cells revealed, at the apical membrane, the presence of circular “pits” measuring 0.5-2 µm in diameter and containing depressions measuring 100-180 nm in diameter^16^. Neither the pits, nor the depressions were identified in the basolateral cell membrane. After the stimulation of secretion, the diameter of depressions transiently increased, returning to the initial size upon completion of cell secretion. Moreover, the exposure of cells to a fungal toxin (cytochalasin B), which inhibits actin polymerization and cell secretion, resulted in a decrease in depression size and a dramatic reduction of secretion. Both these findings suggested that these depressions, which were later called “porosomes” (in a paper submitted in September 2002, and published in February 2003)^14^, are candidates to be considered the secretory portal of the cell. The ultrastructural aspects of the porosomes seen in acinar cells were also confirmed in other cells. For example, other secretory cells have been examined by several researchers and porosomes were identified in chromaffin cells of the adrenal medulla^19^, growth hormone secreting cells of the pituitary gland^20^, neurons^21,22^, astrocytes^23,24^, β-cells of the endocrine pancreas, mast cells^25^, hair cells^26^ and respiratory epithelium^27^. All of the studies on porosome structures in secretory cells have been similar, suggesting that porosomes are universal portals for cell secretion.

In order to confirm that porosomes are indeed the sites where secretory vesicles dock and fuse with the plasma membrane additional studies were needed. Thus, immuno-gold particles were used along with AFM to study the pathways of secretory proteins. In these ultrastructural studies, the antibody-gold particles were found to be located in the depressions or porosomes following stimulation of cell secretion^20,28^.

The morphology of porosomes on the cytoplasmic side or compartment of the cell membrane was also revealed with the help of the AFM. Isolated plasma membranes were imaged in physiological buffer, showing the presence of scattered disks ranging from 0.5 to 1 µm in diameter, with inverted cup-shaped structures inside, corresponding to the pits and depressions or porosomes, respectively^29^. This was also confirmed by immuno-AFM, using antibodies specific to t-SNAREs, which were found to be located at the base of the inverted cup-shaped porosome structures.

Although the discovery of porosomes was facilitated by AFM, images of these structures have also been captured with conventional electron microscopy^16,29,30^. This confirmed the cup-shaped architecture of the porosome, but these studies also offered some new insights. For example, porosomes have a basket-like structure, with three lateral ridges and some vertical ridges. At the base of the cup, there is a ring-like structure^16,29^, considered to be organized by SNAREs and associated proteins.

However, identification of porosome morphology at the nanoscale will have to be reconciled with molecular characterization of these structures in order to construct a more precise model of the porosome.

## 4. Molecular organization of the porosome

Analysis of the porosome composition using proteomic analysis revealed the presence of several proteins: SNAP23/25, syntaxin (t-SNAREs), actin, α-fodrin, vimentin, calcium channels β3 and α1c, the SNARE regulatory protein NSF (N-ethyl-maleimide sensitive factor), and chloride channels ClC2 and ClC3, among others^16,29,31^. Based on the presence of various proteins and their known functions we can propose at least some of their roles in vesicle fusion and secretion. For example, α-fodrin regulates exocytosis through its interaction with the C-terminal region of syntaxin^32^, whereas vimentin filaments interact with SNAP 23/25^33^. Since the administration of the chloride channel blocker DIDS results in blockage of porosome function in the pancreas, the activity of these channels is required for porosome function and secretion^29^. Studies using yeast two-hybrid analysis confirmed the presence and direct interaction of calcium channels with t-SNAREs within the porosome^34^. These channels located at the porosome level could provide the dehydrated calcium ions necessary for membrane fusion.

The fusion proteins in the target membrane, t-SNAREs, interact with v-SNAREs, their partners found in the secretory vesicles, in the presence of calcium ions^35-39^. AFM studies performed on reconstituted lipid bilayers and lipid vesicles showed that t-SNAREs and v-SNAREs from opposing lipid bilayers come in contact to form a ring complex, in the presence of calcium ions^40^. The interactions t-/v-SNARE allow the two lipid bilayers to be very close, at a distance of approximately 2.8 Å^41^. Using light scattering and x-ray diffraction, it was shown that calcium ions bridge the remaining gap between the opposing bilayers, leading to the loss of the ions’ water shell. Thus, the opposing glycerolphospholipids of the two membranes can reassemble, resulting in membrane fusion^40,42,43^.

Porosomes have also been described in nerve synaptic membranes. In neurons, the ring complex is present at the base of the porosome, and is composed of three SNARE pairs^44,45^. Synaptic vesicles transiently dock and fuse with the presynaptic membrane at the level of neuronal porosomes, which are permanent structures^44^. Negative staining of isolated neuronal porosomes and their imaging using EM showed that proteins from the central plug of the complex interact with peripheral porosome proteins. Their arrangement and connections were elucidated with the help of electron density and contour maps, as well as 3D topology profiles^46,47^. As opposed to porosomes from pancreatic acinar cells, neuronal porosomes are much smaller (14-15 nm), and their central channel, which allows the release of neurotransmitter in the synaptic cleft, measures only 1-1.5 nm in diameter. In order for precise fractional release of neurotransmitters from synaptic vesicles, these membrane bounded structures swell due to a regulated entry of ions and water, which leads to a high intravesicular pressure, facilitating the release of neurotransmitter^48,49^. Moreover, the synaptic porosomes responsible for membrane fusion and neurotransmitter release in the synaptic cleft have a 3-4 nm diameter central plug that regulates or induces pore formation under tension created by interactions between t- and v-SNAREs^44,45^. Neuronal porosome assembly was also shown to require cholesterol^50,51^. Syntaxin-1 co-localized with cholesterol in a synaptosomal membrane preparation. Following the administration of saponin, depletion of cholesterol led to the dissociation not only of syntaxin-1, but also of N-type calcium channels from the porosome complex^50^.

## 5. Porosome or fusion pore or porosome and fusion pore

When the porosome was initially defined in the literature, the term was considered as a more appropriate name for the two-word term “fusion pore”^16,52^. The etymology of the name is very suggestive: a permanent nano-body (“soma”/some), at the cell membrane level, organizing a putative pore during the fusion of the membrane of a secretory vesicle with the cell membrane. However, introduction of the new term provoked a controversy. Many scientists working on intracellular membrane trafficking, cell secretion and membrane fusion preferred (and still prefer) to use the term “fusion pore.” Indeed, a literature search in Pub Med from 2003 to August 27, 2014 revealed that 409 publications used the term fusion pore, whereas only 48 publications used the term porosome. It seems that the term “fusion pore” is preferred by physiologists, biochemists, biophysicists who are very active in the research of membrane fusion. It has to be considered that a schism exists between scientists who use the term “fusion pore” to describe a fusion machine that initially was considered to be organized by membrane proteins but now is proposed to be driven by membrane phospholipids^53^. Moreover, some neuroscientists consider the synaptic fusion machines as “active zones” where “synaptic vesicles dock[ed] at specialized portions of the presynaptic plasma membrane^54^. On the other hand, other scientists point out that “small areas of these membranes [that fuse] draw close, molecules on the two surfaces interact, and structural transformations take place. Membrane fusion requires the action of proteins specialized for this task, and these proteins act as a fusion machine”^55^. It is only necessary to consider these fusion machines as preexistent in the target membrane, as Bhanu Jena consistently proved, and the idea of a new structure, discovered at the cell membrane level, and acting in cell secretion, is obvious. Jena names this new structure porosome. Moreover, at the level of the fusion machinery, “at a critical point in this [fusion] process, a fusion pore forms within the membrane contact site and then expands as the spherical vesicle merges with the flat target membrane.”^54^ In a similar manner, the discoverer of the porosome considers a pore to be formed during the secretory event, to allow the secreted material to get out of the secretory vesicle^25^. Therefore, a porosome and a fusion pore complete each other in accomplishing the last event in cell secretion: exocytosis. It is now clear that ‘fusion pore’ is a pore or continuity established as a result of fusion of two opposing bilayers. Hence, the transient fusion of a secretory vesicle membrane at the base of the porosome membrane establishes a ‘fusion pore’ at the porosome base during cell secretion. This aspect should be clear now.

## 6. Porosome: a membrane microdomain

The nuclear pore, a 120-150 nm structure, is a major feature of the nuclear membranes forming the envelope, and it has been named (incorrectly?) as a separate organelle. For example, Günter Blobel once stated in a lecture presented at Athens, during the *33^rd^ FEBS Congress - 11^th^ IUBMB Conference “Biochemistry of Cell Regulation”*, in 2008, that the nuclear pore is the largest organelle of the cell specialized in transport. The dimensions of the nuclear pore are similar (100-150 nm) to the dimensions of porosomes of the exocrine cells of the pancreas as well as various endocrine cells and even mucin-secreting cells of human airway epithelia^56^.

Logic teaches us that it is necessary to first consider the definition of an organelle before assigning this term to nuclear pores and especially to porosomes. To build the definition of the notion organelle we will start from a saying of George Emil Palade in a lecture held at the Institute of Cellular Biology and Pathology, in Bucharest, in one of the summers between 1981 and 1983, when he yearly visited scientists in Romania. During that lecture, Palade said: “Functions must be understood in terms of structures; structures must be understood in terms of chemistry”. To our knowledge now, this sentence seems to be a truism. According to this current knowledge, we may consider as a definition for the term organelle the following sentence: *An organelle is an intracellular element of cell organization with a complex biochemistry, showing a specific morphology and accomplishing definite complex function(s)*. According to this definition, neither the cell membrane, nor any specific structure in the cell membrane, even the porosome, could be considered an organelle. That is because the porosome does not fit the organelle definition only for not being intracellular. Moreover, dimension is not a feature to be considered in cell organelle definition, to come back to the comparison at the beginning of this section.

The similarity in dimensions of nuclear pores and porosomes is not a valid argument in considering terms and categories for porosomes. The most appropriate category for porosomes is that they are membrane microdomains. Membrane microdomains are dynamic, variable, clusters of various proteins and lipids of size from several tens to several hundreds of nanometers in diameter. They facilitate functions of collected biochemical components, to increase the effectiveness of cellular events occurring at the membrane level.

**Figure 1:**
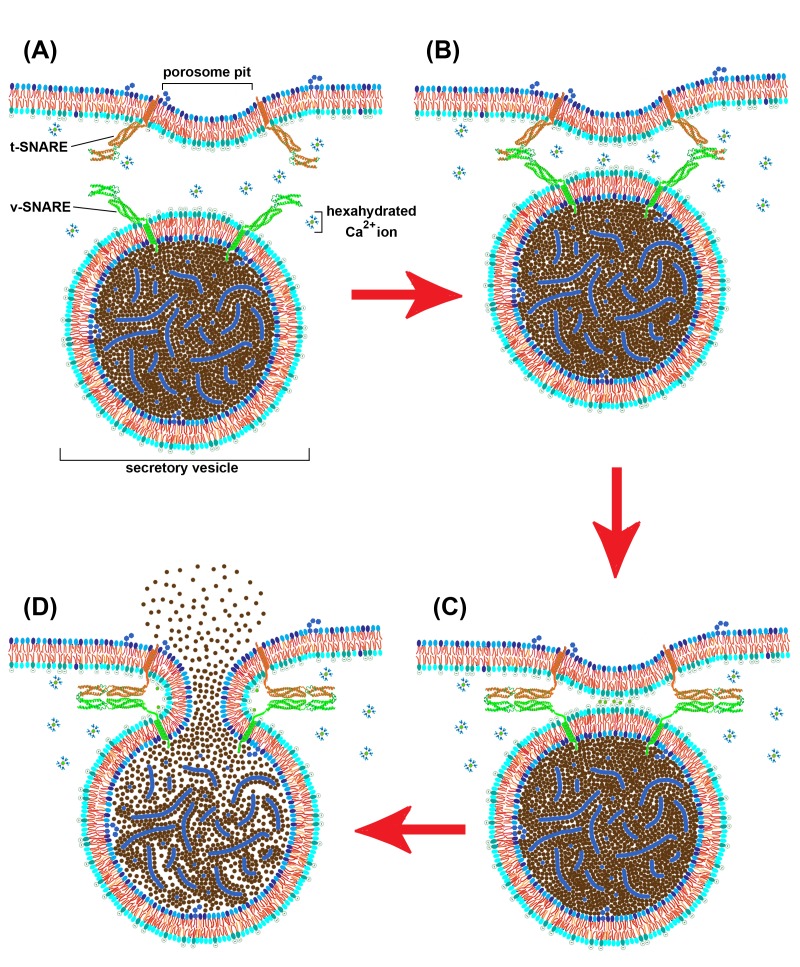
Schematic view of the events facilitated by a porosome in cell secretion (A) A secretory vesicle in a closed apposition to a porosome pit (the molecular complexity of the porosome is not shown, except the docking protein t-SNARE); (B) Vesicle docking by initiation of t-/v-SNAREs interaction; (C) Final interaction of SNARE pairs and dehydrated calcium ions create tensions necessary for membrane fusion; (D) Fusion pore opening and secretion of materials stored inside the secretory vesicle. The blue elements inside the secretory vesicle represent proteoglycans described to be involved in secreting materials’ storage, condensing and exocytosis regulation for many cells^69-74^.

Although lipid-protein membrane microdomains were proposed as early as 1976^57^ and isolated as early as 1988^58^, they have not come into careful focus until the last few years^59-61^. The first described membrane microdomains were lipid rafts (see as a comprehensive review in ^59^) and very soon caveolae were considered as examples of membrane microdomains. Many other membrane microdomains were identified at the cell membrane and/or endomembranes level, assuring a huge diversity of processes such as membrane component sorting and directional membrane trafficking, cell signaling, cell adhesion, and membrane fusion, as well^3,62-64^. Current notions on plasma membrane microdomains have them organized as nano-sized structures present in a variety of cellular membranes^65,66^. Data have now accumulated on particular porosomes from several cellular sources (**Table 1**). These data indicate that porosomes possess all of the features necessary to be described as membrane microdomains. Therefore, porosomes are membrane microdomains and a schematic view about the exocytosis mechanism facilitated by this membrane fusion machine is shown in **Figure 1**.

**Table 1:** Chronology of porosome discovery and characterization

No.	Year	Progress in knowledge	Reference
1	1997	Morphological identification of the structure by AFM in pancreatic acinar cells	^17^
2	2000	Morphological identification of the structure by AFM in neurons	^22^
3	2002	Morphological identification of the structure by AFM in neuro-endocrine cells	^19,20^
4	2002/2003	Coining of the term "porosome"	^19,67^
5	2003	Morphological characterization of the cytosolic side of the porosome by AFM	^16^
6	2003	Characterization of the porosome by TEM	^16,29^
7	2003	Identification of proteins from the porosome complex	^29^
8	2006	Highlighting the importance of cholesterol in the integrity of the neuronal porosome	^50^
9	2008	Creation of 3D EM contour maps of neuronal porosomes	^46^
10	2009	Identification and characterization of porosomes in astrocytes	^23,24^
11	2011	Identification of the porosome complex in the hair cell	^26^
12	2012	Characterzation of the neuronal porosome proteome by chromatography and mass spectrometry	^68^
13	2014	Identification of porosomes in the human airway epithelia by AFM	^27^

## 7. Concluding remarks

More detailed knowledge at the biochemical and biophysical levels are needed to better describe the process of exocytosis (and intracellular vesicle trafficking). We are beyond the debate on whether the terms “fusion pore” or “porosome” best describe the membrane microdomains that constitute the specialized membrane structures of secretion, and it is now time to accept that porosomes are likely to be universal cell portals important in precise cell secretion. It will be equally interesting to see if these specialized membrane domains also have additional activities and cellular properties. Taking into account the molecular organization of the porosome, it could be defined as a membrane microdomain forming a supramolecular machine orchestrating cell secretion with great precision in both space and time.

- **The POROSOME is a membrane microdomain with a complex supramolecular organization**.
- **POROSOMES act as universal secretory portals**.
- **The porosome and fusion pore are two different elementsthat cooperate in the final event of cell secretion or exocytosis**.
- **Understanding the cooperative molecular events between porosomes and fusion pores will advance our knowledge of exocytosis and secretion**.
